# Usefulness of ^18^ F-FDG-PET/CT in aortic graft infection: two cases

**DOI:** 10.1186/1749-8090-9-42

**Published:** 2014-03-05

**Authors:** Eiki Tayama, Hidetsugu Hori, Tomohiro Ueda, Takanori Kono, Ken-ichi Imasaka, Takeaki Harada, Yukihiro Tomita

**Affiliations:** 1 Department of Cardiovascular Surgery, Clinical Research Institution, Kyushu Medical Center, National Hospital Organization, 1-8-1 Jigyohama, Chuo-ku, Fukuoka 810-8563, Japan

**Keywords:** ^18^ F-FDG-PET/CT, Graft infection, Diagnostic imaging

## Abstract

Diagnosis of vascular graft prosthesis infection is crucial, but not straightforward. Here we report two cases in which [^18^ F] fluoro-2-deoxy-D-glucose positron emission tomography/computed tomography (^18^ F-FDG PET/CT) was very useful in the diagnosis of aortic graft infection. Case 1: A 77-year-old Japanese man, two months status post aortic arch graft surgery, suffered from repeated fevers. Blood cultures revealed bacteremia. ^18^ F-FDG-PET/CT ruled out graft infection and diagnosed lumbar pyogenic spondylitis, which was treated with antibiotics, sparing the patient a possible reoperation. Case 2: A 53-year-old Japanese man, seven years status post replacement of the aortic root and ascending aorta, had been suffering from an ostensibly aseptic fistula for over a year and a half. Although repeated CT findings had been negative, ^18^ F-FDG-PET/CT clearly demonstrated communication between the fistula and the ascending aortic graft. He was treated with repeat ascending aortic replacement, omentopexy, and antibiotics. Our experience supports ^18^ F-FDG-PET/CT as a promising modality in cases of suspected vascular graft infection.

## Background

Vascular graft prosthesis infection is a serious complication of vascular surgery. Its diagnosis is often elusive. [^18^ F] fluoro-2-deoxy-D-glucose positron emission tomography/computed tomography (^18^ F-FDG PET/CT) is an established imaging tool for tracing suspected inflammation or infection [1,2]. The fusion of ^18^ F-FDG-PET and computed tomography scans (^18^ F-FDG-PET/CT) acquired in a single session enables precise localization of abnormal ^18^ F-FDG uptake [2]. These properties make it a promising modality for the noninvasive diagnosis of graft infection [1,3-6].

We report two clinical cases where ^18^ F- FDG-PET/CT was very useful to arrive at a definitive diagnosis and to determine proper treatment.

## Case presentation

### Case 1

**Figure 1:**
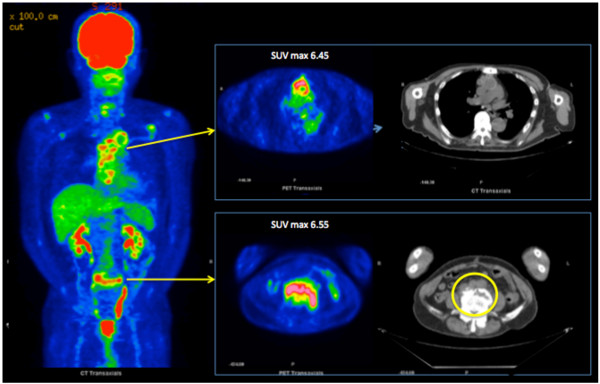
^**18**^ **F-FDG-PET/CT in Case 1.***Circle*; Abnormal uptake of ^18^ F-FDG in the upper part of the 5^th^ lumbar vertebra (SUVmax 6.55); osteolysis was evident at the same site on CT. *; Although substantial ^18^ F-FDG accumulation was seen around the ascending and arch aortic graft, it was judged as physiological.

**Figure 2:**
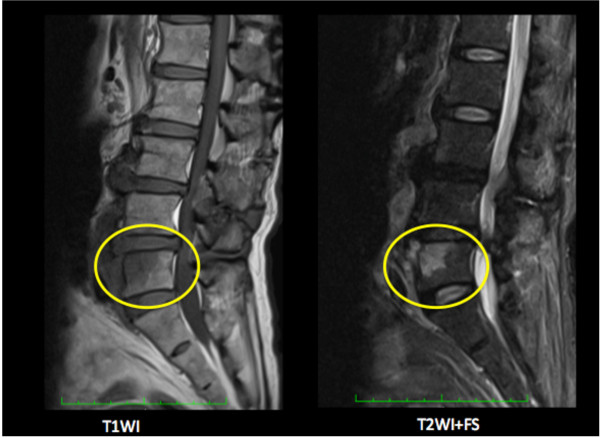
**MRI in Case 1.***Circle*; Low-intensity area with unclear boundary on the T1-weighted image (*left*) and high intensity on the T2-weighted image + fat saturation (*right*) on the ventral side of the 5^th^ lumbar vertebral body.

A 77-year-old Japanese man who underwent total aortic arch replacement for chronic Stanford B aortic dissection was readmitted to our hospital two months later with fever (> 38°C) and shaking chills. Compared with an early postoperative examination, admission chest CT showed no change in low-intensity areas around the ascending portion of the graft without associated fluid or gas. Echocardiography did not show any infectious signs, such as intravascular vegetations or effusion around the graft. *Enterococcus faecalis* grew from admission blood cultures. Because the patient was found to have periodontitis with the same bacteria, tooth extraction was performed and antibiotic treatment (meropenem trihydrate 0.5 g twice a day) instituted for 10 days. He was discharged from hospital with a C-reactive protein (CRP) of 2.26 mg/dL. One month after discharge, he again experienced fever (37.5°C) with increased inflammatory signs. At that time, an implanted tooth was extracted, though it looked normal, under antibiotic coverage. The fever temporarily improved, but increased CRP (> 3 mg/dL) persisted. At 80 days after the initial fever, he was again febrile. Blood cultures demonstrated *Enterocoocus faecalis* again, while the periodontitis had resolved. Because we could not find the fever source with CT or ultrasound, he underwent ^18^ F-FDG-PET/CT, which showed abnormal uptake in the upper part of the fifth lumbar vertebra with a maximum standard uptake value (SUVmax) of 6.55, and corresponding osteolysis at the same site on the CT images (Figure 1). Substantial ^18^ F-FDG accumulation was seen around the ascending and arch aortic graft (SUVmax 6.45), but it was judged as physiological, as its accumulation was diffuse. Lumbar magnetic resonance imaging (MRI) showed a low-intensity area with unsharp margins on T1-weighted images and high intensity on T2-weighted images with fat saturation on the ventral side of L5 (Figure 2). Because the patient had a history of chronic back pain that had not worsened, the physical findings had not been helpful. As a result of the ^18^ F-FDG PET/CT and MRI, L5 pyogenic spondylitis was diagnosed as the source of infection, and long-term antibiotics were administered.

**Figure 3:**
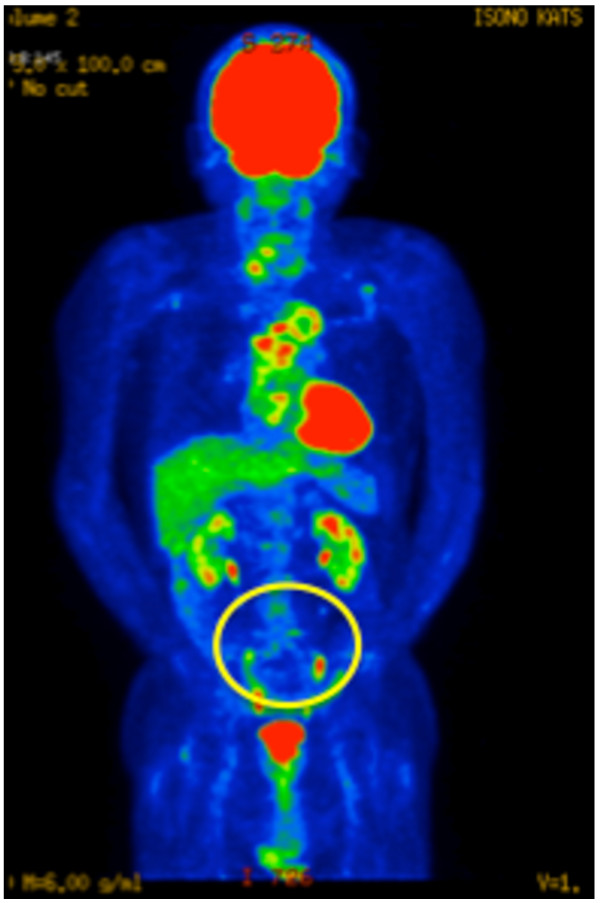
^**18**^ **F-FDG-PET/CT in Case 1, 18 months after treatment for pyogenic spondylitis.** It showed resolution of the abnormal accumulation of ^18^ F-FDG in L5, while both SUVmax (6.43) and the distribution pattern of ^18^ F-FDG around the aortic graft were unchanged. *Circle*; Abnormal accumulation of ^18^ F-FDG in L5 has disappeared.

Eighteen months after the diagnosis, a second ^18^ F-FDG-PET/CT showed resolution of the abnormal accumulation of ^18^ F-FDG in L5, while both SUVmax (6.43) and the distribution pattern of ^18^ F-FDG around the aortic graft were unchanged (Figure 3). The antibiotics were discontinued. He has been doing well for six months after cessation of antibiotic therapy (CRP < 0.03 mg/dL).

### Case 2

**Figure 4:**
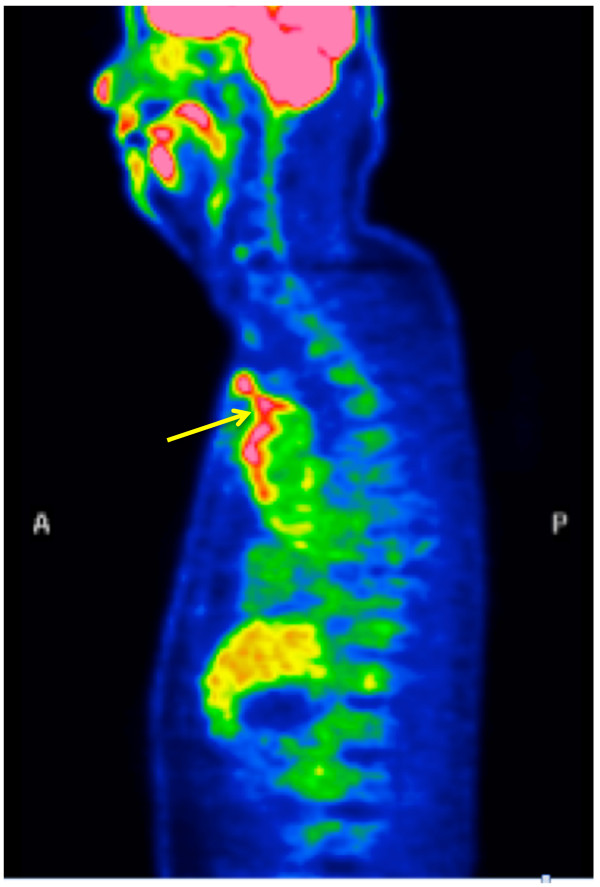
^**18**^ **F-FDG-PET/CT in Case 2.***Arrow*; Abnormal ^18^ F-FDG accumulation is extensive around the ascending aortic graft (SUVmax 6.22), continuous from the upper edge of the graft to the fistula.

A 53-year-old Japanese man seven years status post replacement of the aortic root and ascending aorta (mechanical valve and Dacron graft) for aortic root ectasia, aortic insufficiency, and chronic aortic dissection (Stanford A, DeBakey Type II) reported pain, redness, and bulging around the upper sternotomy site. We diagnosed chronic sternal wire infection and extracted the wires with wound debridement. At that time, thick yellow-white purulent material was observed. It was culture-negative. The pain and redness, leukocytosis, and increased CRP resolved immediately. However, small amounts of culture-negative discharge from the wound persisted, suggesting a fistula. Several chest CTs demonstrated no obvious abnormalities to suggest infection or inflammation around the graft. Despite antibiotic therapy, wound debridement, and vacuum-assisted wound closure therapy, the fistula remained. The patient declined major surgery, because he had already returned to work. Over 500 days after he first presented with pain, he was febrile, despite negative wound and blood cultures. An ^18^ F-FDG-PET/CT examination found abnormal ^18^ F-FDG accumulation around the ascending aortic graft (SUVmax 6.22), extending from the superior aspect of the graft to the skin through the superior margin of the sternum (Figure 4), suggesting fistulous communication with the graft. He underwent a repeat ascending aorta replacement, plus omentopexy, 595 days after the initial presentation with inflammatory signs and symptoms. At surgery, white-yellow purulent material was found around the felt reinforcing the distal anastomosis of the ascending aortic graft. Fortunately, the infection was localized to the distal anastomotic site. The aortic valve prosthesis and aortic root graft prosthesis were left in place. The space around the new graft and anterior mediastinum were filled with a substantial amount of omentum. Methicillin-sensitive *Staphylococcus aureus* was cultured from the removed felt. His postoperative course was uneventful, with discharge from the intensive care unit on postoperative day (POD) 1. The CRP level was below 2 mg/dL on POD 14. He was discharged from the hospital on POD 17. Minomycin hydrochloride 200 mg/day was begun on POD 8 as other intravenous antibiotics were discontinued, and he remains on that dose as an outpatient, because the old aortic valve prosthesis and aortic root graft prosthesis were left in place. His condition has been good without any sign of recurrent infection 10 months after the operation.

## Discussion

Graft infection following prosthetic vascular reconstruction is an uncommon but severe complication. The clinical presentation is sometimes subtle and non-specific and may occur long after surgery. Early diagnosis and treatment are essential to prevent further complications and to avoid unnecessary repeat surgery and removal of uninfected grafts [1,3]. Infection of aortic grafts is generally more serious than that of peripheral arteries, because repeated surgery is so invasive. If prosthetic graft infection is suspected, a patient should be hospitalized, and cultures from both the surgical wound and blood should be taken followed by the initiation of broad-spectrum antibiotics. At the same time, definitive diagnostic imaging is essential. CT scan is the gold standard for assessment of vascular graft infection, though ultrasound scanning and MRI are widely used noninvasive diagnostic modalities [3]. Generally, intact grafts are covered by normal or sometimes fibrotic tissue and are well incorporated into the surrounding tissue. In contrast, infected grafts often appear unattached to the surrounding tissues, and are sometimes surrounded by turbid fluid, air bubbles, or purulent material. It is important to remember that: 1) peri-graft air is present in only about half of all cases, 2) a normal air collections can be seen on CT up to 4–7 weeks following reconstructive surgery [1,7], 3) the presence of lymphocele and non-infected hematoma in the vicinity of the implant hampers diagnosis, 4) low-grade infection also may increase the incidence of false-negative CT studies, 5) the accuracy of CT scans decreases in the chronic phase (acute vascular graft infection is diagnosed with a sensitivity and specificity reaching 100%, but that decreases to 55% in cases of chronic infection) [3]. To increase diagnostic accuracy, repeat examinations over time are often required.

Whereas CT scans afford visualization of the structural changes secondary to infection, nuclear medicine techniques facilitate diagnosis on the basis of molecular biological changes. ^67^Gallium scintigraphy was acknowledged to be a useful examination for inflammation. Unfortunately, it has been shown to be of limited value in the assessment of vascular graft infection, with a relatively low reported sensitivity [1]. While ^99m^Tc- or ^111^In-labeled leukocyte scintigraphy is highly accurate for the detection of infected grafts, other infectious processes localized in the vicinity of the graft can account for false-positive results [1,3].

As opposed to conventional scintigraphy, ^18^ F-FDG-PET/CT has become an established imaging tool. ^18^ F-FDG is a marker of increased intracellular glucose metabolism and is taken up by malignant as well as infectious and inflammatory processes [8]. The diagnostic sensitivity of ^18^ F-FDG/PET-CT is extremely high compared with other diagnostic modalities. In a prospective study including 39 patients with suspected vascular graft infections, ^18^ F-FDG-PET/CT had a sensitivity, specificity, positive predictive value, and negative predictive value of 93%, 91%, 88%, and 96%, respectively [5]. According to Tokuda et al. [9], an SUVmax value of greater than 8 in perigraft tissue is suspicious for graft infection. This cut-off value must be further assessed, as its experience was limited to only nine cases(thoracic graft infections; 4 positive *vs* 5 negative cases). Actually in our Case 2, a definite graft infection, SUVmax around the graft was lower than 8.

The whole-body imaging capabilities of ^18^ F-FDG-PET/CT allow the detection of infectious foci distant from the graft. Faster examination time than conventional scintigraphy required is an additional clinical advantage of ^18^ F-FDG-PET/CT, with preliminary results available within 2 hours [10].

One of the drawbacks of ^18^ F-FDG-PET/CT is its relatively low specificity in infection/inflammation imaging [1,4,10,11]. Fukuchi et al., using PET alone, showed that ^18^ F-FDG-PET had a sensitivity of 91% but a specificity of only 64%, as compared with CT, which had a lower sensitivity of 64% but a specificity of 86% [4]. The early phase of wound healing is physiologically accompanied by an increased accumulation of ^18^ F-FDG [5,12]. Extensive linear FDG uptake of mild-to-moderate intensity along vascular grafts that have no evidence of infection has been previously described [1,4]. Venous thrombosis, sterile inflammation, vasculitis, chronic polyarthritis, or retroperitoneal fibrosis may all lead to an abnormal accumulation of ^18^ F-FDG.

^18^ F-FDG-PET/CT enabled us to find an infectious nidus remote from the graft in Case 1 and to diagnose aortic graft infection in Case 2. Graft infection could have preceded and, in fact, caused the lumbar pyogenic spondylitis in Case 1, as recurrent bacteremia with the same microorganism suggested an intravascular focus. But we speculate that it was an extraaortic source due to the lack of change in the appearance of the graft between the first and second ^18^ F-FDG-PET/CT studies. Regardless, ^18^ F-FDG-PET/CT enabled the diagnosis of an infectious focus, which could then be addressed.

## Conclusion

^18^ F-FDG-PET/CT is able to provide clinically significant information on the origin of infections, including infections of vascular grafts. If aortic graft infection is suspected, we recommend ^18^ F-FDG-PET/CT for definitive diagnosis. This noninvasive diagnostic tool may help to avoid unnecessary surgery and lead to timely, appropriate treatment.

## Consent

Written informed consent was obtained from the both patients for publication of this Case Report and any accompanying images. A copy of the written consent is available for review by the Editor-in-Chief of this journal.

## Abbreviations

18 F−FDG PET/CT: [^18^ F] fluoro-2-deoxy-D-glucose positron emission tomography/computed tomography; CRP: C-reactive protein; L5: The fifth lumbar vertebra; SUVmax: Maximum standard uptake value; MRI: Magnetic resonance imaging; POD: Postoperative day.

## Competing interests

The authors declare that they have no competing interests.

## Authors’ contributions

ET wrote the draft of the manuscript. HH and UT obtained the data and written consent. KT, TH and YT performed the literature review and participated in the manuscript writing of the paper. All authors have read and approved the final manuscript.

## References

[B1] Keidar Z Nitecki S FDG-PET for the detection of infected vascular grafts Q J Nucl Med Mol Imaging 2009 9 35 40 19182726

[B2] Chacko TK Zhuang H Nakhoda KZ Moussavian B Alavi A Applications of fluorodeoxyglucose positron emission tomography in the diagnosis of infection Nucl Med Commun 2003 9 615 624 10.1097/00006231-200306000-00002 12766596

[B3] Legout L D’Elia PV Sarraz-Bournet B Haulon S Meybeck A Senneville E Leroy O Diagnosis and management of prosthetic vascular graft infections Médecine et maladies infectieuses 2012 9 102 109 10.1016/j.medmal.2012.01.003 22341664

[B4] Fukuchi K Ishida Y Higashi M Tsunekawa T Ogino H Minatoya K Kiso K Naito H Detection of aortic graft infection by fluorodeoxyglucose positron emission tomography: comparison with computed tomographic findings J Vasc Surg 2005 9 919 925 10.1016/j.jvs.2005.07.038 16275448

[B5] Keider Z Engel A Hoffman A Israel O Nitecki S Prosthetic vascular graft infection: the role of 18FDG PET/CT J Nucl Med 2007 9 1230 1236 10.2967/jnumed.107.040253 17631553

[B6] Bruggink JLM Glaudemans AWJM Saleem BR Meerwaldt R Alkefaji H Prins TR Slart RHJA Zeebregts CJ Accuracy of FDG-PET/CT in the diagnostic work-up of vascular prosthetic graft infection Eur J Vasc Endovasc Surg 2010 9 348 354 10.1016/j.ejvs.2010.05.016 20576451

[B7] Orton DF LeVeen RF Saigh JA Culp WC Fidler JL Lynch TJ Goertzen TC McCowan TC Aortic prosthetic graft infections: radiologic manifestations and implications for management Radiographics 2000 9 977 993 10.1148/radiographics.20.4.g00jl12977 10903688

[B8] De Winter F Vogelaers D Gemmel F Dierckx RA Promising role of 18-F-fluoro-D-deoxyglucose positron emission tomography in clinical infectious diseases Eur J Clin Microbiol Infect Dis 2002 9 247 257 10.1007/s10096-002-0708-2 12072934

[B9] Tokuda Y Oshima H Araki Y Narita Y Mutsuga M Kato K Usui A Detection of thoracic aortic prosthetic graft infection with 18 F-fluorodeoxyglucose positron emission tomography/computed tomography Eur J Cardio-Thorac Surg 2013 9 1183 1187 10.1093/ejcts/ezs693 23333838

[B10] Stumpe KD Dazzi H Chaffner A Von Schulthess GK Infection imaging using whole-body FDG-PET Eur J Nucl Med 2000 9 822 832 10.1007/s002590000277 10952494

[B11] Spacek M Belohlavek O Votrubova J Sebesta P Stadler P Diagnosis of “non-acute” vascular prosthesis infection using 18 F-FDG PET/CT: our experience with 96 prostheses Eur J Nucl Med Mol Imaging 2009 9 850 858 10.1007/s00259-008-1002-z 19107480

[B12] Cook GJR Fogelman I Maisey MN Normal physiological and benign pathological variants of 18-fluoro-2-deoxyglucose positron-emission tomography scanning; potential for error in interpretation Semin Nucl Med 1996 9 308 314 10.1016/S0001-2998(96)80006-7 8916319

